# Plasma Cytokines for the Prediction of the Effectiveness of TNFα Inhibitors Etanercept, Infliximab, and Adalimumab in the Treatment of Psoriasis

**DOI:** 10.3390/jcm13133895

**Published:** 2024-07-02

**Authors:** Arfenya Karamova, Ludmila Znamenskaya, Anastasiia Vorontsova, Olga Obraztsova, Alexandr Nikonorov, Eugenia Nikonorova, Dmitry Deryabin, Alexey Kubanov

**Affiliations:** 1 State Research Center of Dermatovenereology and Cosmetology, Moscow 107076, Russia; karamova@cnikvi.ru (A.K.); znaml@cnikvi.ru (L.Z.); valeeva19@gmail.com (O.O.); dgderyabin@yandex.ru (D.D.); alex@cnikvi.ru (A.K.); 2 All-Russian Scientific Research Institute of Medicinal and Aromatic Plants (VILAR), Moscow 117216, Russia; gatiatulinaer@gmail.com

**Keywords:** psoriasis, plasma cytokines, TNFα inhibitors, etanercept, infliximab, adalimumab

## Abstract

**Background/Objectives:** Psoriasis is a chronic, inflammatory, immuno-mediated cutaneous disease characterized by a prominent TNFα-IL23/IL17 immune axis. In recent years, targeted therapies have become standard practice for managing moderate-to-severe psoriasis and have demonstrated efficacy. At the same time, identifying factors associated with the success or failure of TNFα inhibitor therapy remains one of the most difficult aspects in psoriasis treatment. **Methods:** A clinical, non-randomized study was conducted to evaluate the impact of TNFα inhibitors on the plasma cytokine profiles in patients with moderate-to-severe psoriasis vulgaris (ICD-10 code L40.0). The patients were treated with either etanercept, adalimumab, or infliximab for 16 weeks. Plasma cytokine profiles were assessed using a BioPlex200 System. **Results:** By the 16th week of therapy, a positive treatment response (PASI ≥ 75) was observed in 51 patients (63%), while 30 patients (37%) showed no response (PASI ≤ 50). When using etanercept, a positive effect was observed in 11 patients (41%), in 14 patients (52%) using adalimumab, and in 26 patients (96%) using infliximab. Analysis of the baseline cytokine levels revealed no differences between the “positive effect” and “no effect” groups, except for IL20, which was 2.61 times higher in the “positive effect” group compared to the “no effect” group, suggesting its potential predictive role in the effectiveness of therapy with TNFα inhibitors. Treatment led to a decrease in IL17F, IL31, sCD40L, and VEGF for all patients, and in IL20 for the “positive effect” group. The increase in ICAM1 in the “no effect” group suggests the possible retention of active migration and the fixation of T cells in the affected skin in these patients. No significant difference in cytokine levels was observed when categorizing patients into subgroups based on the effectiveness of therapy with etanercept, infliximab, and adalimumab; only a pre- and post-treatment difference in the whole cohort was noted. A random forest model showed the importance of VEGF, sCD40L, and ICAM1. **Conclusions:** The baseline levels of VEGF, sCD40L, and ICAM1, as well as IL20, could serve as potential predictors of treatment effectiveness using TNFa inhibitors. However, this hypothesis requires confirmation with a larger patient population.

## 1. Introduction

Psoriasis is a chronic, inflammatory, immuno-mediated cutaneous disease characterized by a prominent TNFα-IL23/IL17 immune axis [1]. Considering the pivotal role of TNFα in the pathogenesis of immunoinflammatory diseases, there are several FDA-approved anti-TNFα medications available for the treatment of psoriasis, including infliximab, adalimumab, certolizumab pegol, golimumab, and etanercept [2]. In recent years, targeted therapies, particularly TNFα and IL17 inhibitors, have become standard practice for managing moderate-to-severe psoriasis and have demonstrated efficacy [3,4]. However, despite TNFα inhibitors being the preferred pharmacological intervention for immune-mediated inflammatory diseases, approximately 30–40% of patients experience “primary therapeutic failure” or a loss of response over time [4,5,6,7]. Therefore, significant efforts are being made to identify biomarkers that can predict the suitability of TNFα inhibitors in patients [2].

It has been shown that low baseline levels of S100A12 and prealbumin together with high platelet factor 4 may predict the response to anti-TNFα drugs such as adalimumab, infliximab, and etanercept in patients with RA [8]. A systematic review involving patients with psoriatic arthritis and psoriasis has indicated that serum levels of IL12 and single nucleotide polymorphisms (SNPs) in the IL12B gene are promising biomarkers for predicting the response to anti-TNFα treatment [9]. Andersen et al. conducted a study which demonstrated that patients who responded to adalimumab treatment had lower initial levels of IL6 in their bloodstream compared to non-responders (0.99 (0.42–1.4) vs. 1.62 (0.96–2.41) pg/mL; *p* = 0.02), suggesting that IL6 could potentially serve as a prospective marker for predicting the response to TNFα inhibitors in psoriasis patients [10]. However, the authors also noted that concentrations of IL17A, IL1β, and IFNγ were mostly below the quantification limits [10], which complicates the use of cytokines as prognostic markers. Currently, there are no definitive diagnostic indicators that can be employed to predict the response to TNF inhibitors therapy, as the findings from numerous studies are highly contradictory [2]. Given these challenges, identifying factors associated with the success or failure of TNFα inhibitor therapy remains one of the most difficult aspects in psoriasis treatment [11].

Therefore, further investigation is needed to discover immunological predictors of treatment effectiveness. The objective of this study was to evaluate plasma cytokine levels and identify potential predictors of treatment effectiveness with TNFα inhibitors in patients with moderate-to-severe psoriasis. In our article, we showed for the first time that the baseline levels of cytokines VEGF, sCD40L, ICAM1, and IL20 may serve as predictors of treatment efficacy with TNFα inhibitors. Unlike other cytokines, which play an important role in the pathogenesis of psoriasis and had a relatively small percentage of non-zero values in plasma, the proposed predictors were identified in a significant percentage of cases. 

## 2. Materials and Methods

The present study enrolled 81 patients diagnosed with moderate-to-severe psoriasis vulgaris (L40.0, ICD-10), aged between 19 and 76 years (mean age 46.56 ± 13.69 years), comprising 63 (78%) men and 18 (22%) women. The patients were treated with either etanercept (Enbrel^®^, Pfizer, Bruxelles, Belgium), adalimumab (Humira^®^, Vetter Pharma-Fertigung, Ravensburg, Germany), or infliximab (Remicade^®^, MSD International GmbH, Singapore). The study was approved by the Local Ethics Committee (protocol N° 04, 27 April 2018) and adhered to the standards of good clinical practice and evidence-based medicine. All patients provided written informed consent to participate in the study. The exclusion criteria included the presence of symptoms suggestive of tuberculosis, viral hepatitis B and C, HIV infection, syphilis, other autoimmune diseases, and standard contraindications to the use of etanercept, infliximab, and adalimumab [12]. An additional inclusion criterion was the failure or intolerance to previous therapy (phototherapy, therapy with methotrexate, or acitretin) [12].

To evaluate the severity of psoriasis and the efficacy of the therapy, the study employed the psoriasis area and severity index (PASI), body surface area (BSA), and static physician global assessment (sPGA) scores. The severity of psoriasis was classified as moderate (10 ≤ PASI < 20) or severe (PASI ≥ 20). Clinical effectiveness was assessed as a decrease in PASI score (in %) after the treatment, using the formula: (PASI before the treatment − PASI after the treatment)/PASI before the treatment * 100%. The efficacy of the treatment was assessed at week 16 and patients were categorized into a positive effect group (PASI ≥ 75, good and satisfactory effect) or a no effect group (PASI ≤ 50, bad effect). Further analysis was conducted on these groups.

The physical examination of patients with moderate-to-severe psoriasis and plasma sampling were performed by employees of the State Research Center of Dermatovenerology and Cosmetology. The baseline and week 16 plasma cytokine profiles were assessed using a BioPlex200 System (Bio-Rad, Hercules, CA, USA) which is a suspension array system that allows to perform multiplex assay of up to 100 biomolecules in a single sample. Procarta immunoassay Human Mag 9 plex One PL (PC1009M) kit (Thermo Scientific, Norristown, PA, USA), Bio-Plex Pro Human Cytokine ICAM-1 (171B6009M) Plex (Bio-Rad, Hercules, CA, USA), and Bio-Plex Pro Human Th17 Cytokine Panel 15-Plex (Bio-Rad, Hercules, CA, USA) kit, with the corresponding calibration kit were used for analysis; the obtained results were expressed in pg/mL. All stages were carried out according to the manufacturer’s instructions, under the same conditions for all samples. Cytokines with concentrations below the assay working range were assigned a zero value.

The analysis and visualization of data were carried out using RStudio and the R programming language (version 4.2.2) [13]. Data distribution was evaluated using the Shapiro–Wilk test. Data were presented as mean ± standard deviation/standard error of mean and medians and quartiles (Me (Q1–Q3)) depending on the data distribution. The difference between three or more groups characterized by Gaussian distribution was assessed using one-way ANOVA followed by a Tukey post hoc test for multiple comparisons. The two-sample Wilcoxon test for independent samples was used to compare two groups with non-Gaussian distribution, and the Kruskal–Wallis test followed by a pairwise Wilcoxon test with Bonferroni correction for multiple testing was used for comparing three or more groups. The paired samples Wilcoxon test was used for pairwise comparisons before/after treatment. Differences were considered significant at *p* < 0.05.

The search for relationships between the baseline levels of cytokines and the effectiveness of therapy included a preliminary analysis of the data to exclude multicollinearity (by computing a Spearman correlation matrix). The development of the model included a random forest (RF) model with data splitting into training and test samples in the ratio of 80% and 20% using the factor as an argument of the createDataPartition function of the “caret” package [14]. The RF model was trained using the randomForest function of the “randomForest” package, with the parameters ntree = 500, mtry = 18 (equal to the number of predictors) [15]. After fitting the model on the training sample with the specified parameters, the response was predicted on the test sample with an evaluation of the model metrics (accuracy, specificity, sensitivity, area under the ROC curve) and an evaluation of the importance of predictors.

## 3. Results

### 3.1. General Characteristic of Patients

All patients were divided into three groups and received different TNFα inhibitors (etanercept, infliximab, and adalimumab). The clinical data of patients are shown in Table 1. Moderate and severe psoriasis was diagnosed in 39 (48%) and 42 (52%) patients, respectively. There were differences in age between patients treated with infliximab and etanercept (*p* = 0.010), whereas no differences were found in BMI. Baseline PASI, BSA, and sPGA values were significantly different between the studied groups. The values of PASI scores recorded during the initial examination ranged from 10.3 to 64.2, the BSA score ranged from 11 to 90, sPGA ranged from 2 to 4 (Table 1). PASI in the group treated with infliximab was 47.5% higher than that in the etanercept group (*p* < 0.001) and 84.8% higher than that in the adalimumab group (*p* = 0.001). BSA in the infliximab group was 2.25 and almost 2-fold higher than that in the etanercept (*p* < 0.001) and adalimumab group (*p* < 0.001), respectively. The same differences were observed in sPGA score: it was 1,4- and 1,33-fold higher than that in the etanercept (*p* < 0.001) and adalimumab group (*p* = 0.005), respectively. This fact is explained by considering the route of administration of the drug, possible side effects, and the effectiveness of the therapy when prescribing these drugs by a dermatologist.

Table 1 also illustrates the treatment outcomes of the patients. It can be observed that the 11 patients (41%) treated with etanercept, 14 patients (52%) treated with adalimumab, and 26 patients (96%) treated with infliximab achieved a PASI score of ≥75. Overall, a positive treatment effect was observed in 51 patients (63%), while no effect was seen in 30 patients (37%).

### 3.2. Comparative Analysis of Cytokine Levels Depending on the Effectiveness of Therapy with TNFα Inhibitors

Taking into account that a substantial number of patients exhibited cytokine levels falling below the limit of quantitation for the majority of variables studied (Table 2), descriptive statistics are shown only for non-zero values (see Table 3).

Statistical analysis of baseline cytokine levels between patients with positive and negative effects of therapy did not reveal any significant differences between the study groups except IL20 (Table 4). Thus, the baseline level of IL20 in the positive effect group significantly exceeded the levels observed in the no effect group by a factor of 2.61 (*p* = 0.045). Treatment with TNFa inhibitors led to a significant 2.2-fold reduction in IL20 levels in positive effect group, whereas in the non-responders, IL20 concentrations remained unchanged.

Pairwise comparison of cytokine levels before and after treatment showed a significant decrease in IL17F (*p* = 0.008), IL31 (*p* < 0.001), sCD40L (*p* < 0.001), and VEGF (*p* < 0.001), and an increase in ICAM1 (*p* = 0.002). At the same time, alterations in IL31, sCD40L, and VEGF levels were observed across all patients irrespective of treatment response, while ICAM1 levels exhibited changes solely in the no effect group.

### 3.3. Comparative Analysis of Cytokine Levels Depending on the Drug Used

There was significant difference in VEGF levels between the drug used both prior to treatment (*p* = 0.045) and post-treatment (*p* = 0.013) as determined by the Kruskal–Wallis test. Subsequent pairwise comparisons, adjusted for multiple comparisons, revealed differences in VEGF concentrations between Enbrel and Remicade-treated groups before (*p* = 0.045) and after treatment (*p* = 0.012).

**Figure 1:**
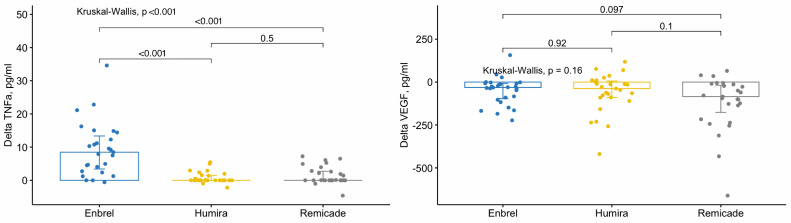
Changes in the level of TNFα and VEGF during the treatment depending on the drug used.

However, when comparing the VEGF deltas (Figure 1) no differences were identified, suggesting comparable alterations during treatment with distinct medications.

The level of TNFa was significantly different only after the treatment (*p* < 0.001), particularly between Enbrel and Remicade- (*p* = 0.005) and Enbrel and Humira-treated groups (*p* < 0.001). Despite this, Enbrel-treated group exhibited the worst treatment response rate, accompanied by an elevation in TNFa levels after the therapy. Additionally, TNFa deltas were markedly different compared to the other two drugs (Figure 1).

Upon categorizing patients into subgroups based on the effectiveness of therapy with etanercept, infliximab, and adalimumab, no significant cytokines were identified as reliable predictors of treatment efficacy with a specific drug.

### 3.4. A Search for Relationships between Baseline Levels of Cytokines and the Effectiveness of Therapy

The subsequent stage of the study involved the development of a model to establish the relationship between baseline cytokine levels and treatment effectiveness. A preliminary analysis of the correlations was conducted to search for multicollinearity. Two predictors with strong correlations (IL33 and IL4 (r = 0.830, *p* < 0.001) were identified and excluded from further analysis. The search for uninformative predictors that exhibited linear dependencies (carrying duplicate information) did not yield any results.

**Figure 2:**
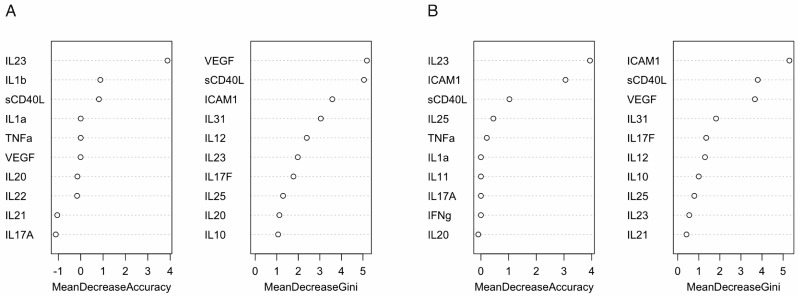
The top 10 significant variables for prediction of the effectiveness of TNFα inhibitors therapy effectiveness evaluated by mean decrease Gini and mean decrease accuracy. (**A**) Model with all drugs; (**B**) Model without Remicade.

In order to more accurately predict the effectiveness, a random forests (RF) model was chosen. After data preprocessing, the entire dataset was divided into training (80%) and testing (20%) samples, and the RF model was built. The metrics of the final model were as follows: accuracy = 0.63, specificity = 0.80, and sensitivity = 0.33. The area under the ROC curve (AUC) was 0.968, which was considered as a good result (Figure 2A) [16].

Based on the results of RF model, 10 significant variables were obtained (Figure 2A), with the most important predictors located at the top of the graph. As can be seen from Figure 2A, the most important variables were IL23 based on the mean decrease accuracy, while VEGF and sCD40L were identified as important predictors based on the mean decrease Gini. At the same time, after excluding data on the drug Infliximab (Remicade) from the model, since it had only one patient with no effect and this could affect the results and accuracy of the model, the most important predictors ICAM1, VEGF, and sCD40L were preserved (Figure 2B). The accuracy and sensitivity of the model increased to 0.7 and 0.80, respectively, and the specificity decreased to 0.60. The area under the curve was 0.979.

## 4. Discussion

The advent of biological therapies has ushered in a new era of effective management of psoriasis [4]. Our study involved 81 patients with moderate-to-severe psoriasis who received targeted therapy with TNFα inhibitors for 16 weeks. The results demonstrated a positive effect of treatment in 51 (63%) patients and no effect in 30 patients (37%). The therapeutic efficacy of etanercept, adalimumab, and infliximab varied, which is consistent with the existing literature data, since each of these TNFα inhibitors has a distinct structure and mechanism of action [17,18]. Etanercept, a fusion protein engineered from human receptors, is structurally distinct from adalimumab and infliximab, which are monoclonal antibodies against human TNFα. Scallon et al. suggest that etanercept is less immunogenic than infliximab due to the formation of unstable complexes with TNFα, resulting in a less pronounced therapeutic effect [19]. Our study confirmed this fact, as the effectiveness of etanercept among the three TNFα inhibitors studied was minimal, and the 16-week incidence of therapeutic failure was 59%. It should be noted that etanercept is safest when used in elderly patients with severe psoriasis [20].

The analysis of cytokine levels and dynamics during treatment with TNFα inhibitors yielded rather complex results. Pairwise comparison of cytokine levels before and after treatment showed a significant decrease in IL17F, IL31, sCD40L, and VEGF, and an increase in ICAM1. The mentioned data were also confirmed by significant variables selected in the RF model for prediction of the effectiveness of the TNFα inhibitors therapy. According to the literature, there is no clear understanding in the selection of important predictors when building an RF model, and each of the methods has its drawbacks. Many biological studies analyze relatively small datasets, so it is often recommended to use the mean decrease Gini, as calculating the mean decrease accuracy using “out-of-bag (OOB) samples” with small samples will yield overly detailed results [21]. For instance, Yu et al. employed the mean decrease Gini to assess imaging biomarkers in predicting the stage of non-small cell lung cancer [22]. In a study by Lloyd et al., this index was also used to predict effective biomarkers of recent and remote infections with *Mycobacterium tuberculosis* [23]. However, in the metabolomic analysis of dogs’ sera with type I diabetes mellitus, the authors used mean decrease accuracy, despite the small sample size [24]. In the present study, an analysis of the RF model data revealed differences in the feature selection for the predictors of the treatment efficacy depending on whether mean decrease accuracy or mean decrease Gini was used for ranking. As we observed variability in the predictors examined by mean decrease accuracy, we identified VEGF, ICAM1, and sCD40L as important predictors of treatment efficiency. Even after excluding data on the drug Infliximab (Remicade) we saw consistency in these predictors.

VEGF is a crucial factor in angiogenesis, and its overexpression and increased presence in the blood have been reported in numerous studies on psoriasis [25,26]. Angiogenesis disorders significantly contribute to the pathogenesis of psoriasis, in addition to keratinocyte dysfunction and immune cell recruitment. These disorders are characterized by the expansion, tortuosity, and increased permeability of capillaries in the dermal papilla [27]. The VEGFA gene, located in the PSORS1 locus, is highly polymorphic and associated with the risk of psoriasis development [28,29], with early manifestation occurring up to age 40 [30]. Furthermore, VEGFA inhibitors are among the promising new drugs for psoriasis treatment [31]. The observed reduction in VEGF after therapy aligns with data on improvements in the condition of the vascular network after treatment with etanercept [32] and infliximab [33].

It is noteworthy that, given the wide heterogeneity of psoriasis pathogenesis, vascular growth and VEGF activity may be the most universal mechanisms. In this study, non-zero levels of VEGF were detected in 100% of the plasma samples, which also confirms its diagnostic value. VEGF is linked with other cytokines and factors involved in the pathogenesis of psoriasis. It is believed that VEGF can increase the expression of ICAM-1 in the K14-VEGF transgenic mouse model; using a potent VEGF antagonist normalized the increased ICAM1 expression in the basal keratinocytes and the vasculature [34]. In a study by Wen et al., applying gambogic acid (an angiogenesis inhibitor) on the K14-VEGF transgenic model resulted in a significant decrease in both the expression of VEGF receptor 2 and its signaling pathway [35]. In our view, the observed increase in ICAM-1 levels in the total group and no effect group may reflect a slower rate of ICAM-1 decline compared to the positive effect group. TNFα inhibitors can simultaneously reduce ICAM-1 levels while reducing VEGF. Moreover, ICAM-1 did not differ from the baseline in the positive effect group, indicating effective inhibition of VEGF and ICAM-1 production in these patients.

Furthermore, the levels of IL17F, IL31, and CD40L (which was identified as a significant predictor), decreased from baseline after treatment and did not differ between the drugs administered. In a study by Venerito et al., a marked reduction in sCD40L levels was observed in patients with psoriatic arthritis treated with ampremilast, leading the authors to conclude that it can predict the clinical response to ampremilast therapy [36]. In our previous investigation, the levels of sCD40L and IL31 (as well as IL23 and IL25) in lesional skin were significantly reduced by weeks 14 and 26 of treatment with ampremilast, while other cytokines initially decreased at week 14 and then inexplicably increased [37]. IL17F is undoubtedly a crucial component in the pathogenesis of psoriasis as part of the TNFα—IL23/IL17 axis [17], and its decrease during TNFα inhibitor therapy is a logical consequence of TNFα suppression. The comparison of the delta TNFα and TNFα levels at week 16 revealed that the highest TNFα levels and delta were observed in patients treated with etanercept. This fact is consistent with the worst efficacy of etanercept in the present study and with existing data on the instability of its complexes with TNFα and lower efficacy compared to other drugs [19].

There were also a higher baseline IL20 levels in patients with a positive effect compared to those with a negative effect of therapy. In the current concept of psoriasis immunopathogenesis, immune cells (keratinocytes, pDCs, macrophages, NKT cells) activated by triggers start to produce INFγ, TNFα, IL1β, and IL6, which activate mDC [38]. Those, in turn, stimulate the differentiation and proliferation of different types of T1, T17, and T22 lymphocytes. The pathogenetic TNFα—IL23/IL17 axis leads to an increased production of IL22 and IL17A/F, affecting keratinocyte proliferation and differentiation, causing characteristic psoriatic lesions [38,39]. The IL20 belongs to the IL10 family with IL22 and possesses pro-inflammatory properties. It was shown that its expression increased in lesional psoriatic skin [40]. This cytokine was included in the top 10 predictors but with a lesser level of importance. Thus, an increased level of IL20 may be a predictor of the effectiveness of therapy, and one of the effects of TNFα inhibitors may be a decrease in the level of IL20, which mediates local inflammation. However, this assumption needs to be confirmed with a larger sample size.

The limitation of this study was the limited number of participants, which precluded the identification of possible differences when comparing the drugs administered. Additionally, the analysis was complicated by a large number of patients with multiple cytokines below the detection limit.

In conclusion, this study yields several noteworthy findings:

1. Remicade (infliximab) demonstrated a maximal efficacy (96%) in the treatment of psoriasis using TNFa inhibitors.
2. Comparison of non-zero baseline cytokine levels based on treatment efficacy did not reveal significant differences, except for IL20, which exhibited a 2.61-times higher concentration in the positive effect group compared to the group with no effect. This observation suggests that IL20 could serve as a potential predictor of treatment effectiveness with TNFa inhibitors.
3. Upon comparing pre- and post-treatment non-zero cytokine levels, a reduction in IL17F, IL31, sCD40L, and VEGF was observed in all patients, along with a decrease in IL20, specifically in the positive effect group. These findings indicate a decline in pro-inflammatory processes and potential stimulation of vascular growth in affected skin, as supported by clinical evidence (decreased PASI scores). Conversely, elevated ICAM1 levels in the no effect group may reflect an ongoing T cell migration and retention in affected skin, contributing to persistent local inflammation in psoriasis.
4. Upon categorizing patients into subgroups based on the effectiveness of therapy with etanercept, infliximab, and adalimumab, no cytokines were identified as reliable predictors of treatment efficacy with a specific drug.
5. The exploration for the key predictors of treatment efficacy using a random forest model showed the importance of baseline levels of VEGF, sCD40L, and ICAM1 across all patients included in the model, even after excluding those receiving Remicade.

## 5. Conclusions

The results of the study suggest that baseline levels of cytokines VEGF, sCD40L, and ICAM1, as well as IL20, could serve as potential predictors of treatment effectiveness using TNFa inhibitors in patients with psoriasis. However, further studies with a larger patient cohort are necessary to confirm this hypothesis.

## Figures and Tables

**Table 1:** Data of patients with moderate-to-severe psoriasis treated with selective TNFα inhibitors and efficacy of TNFα inhibitors assessed at week 16.

	Total Group (n = 81)	Etanercept (n = 27)	Adalimumab (n = 27)	Infliximab (n = 27)
Age	46.52 ± 13.69/1.53	51.15 ± 14.46/2.78	48.12 ± 13.63/2.67	40.48 ± 10.93/2.10
BMI	27.72 ± 4.8/0.57	27.99 ± 4.53/0.92	28.43 ± 4.62/0.98	26.89 ± 5.28/1.08
PASI score	21.0 (13.6–29.0)	15.8 (12.55–21.55)	19.8 (12.41–24.6)	29.2 (22.95–42.5) ^1,2^
BSA	25.5 (16.0–37.0)	20 (14.5–26.5)	23 (15.5–28.5)	45 (27.5–68) ^1,2^
sPGA	2.79 ± 0.89/0.10	2.41 ± 0.57/0.11	2.56 ± 0.65/0.12	3.41 ± 1.05/0.20 ^1,2^
The severity of psoriasis before the treatment				
Moderate	38	19	14	5
Severe	43	8	13	22
Positive effect of treatment				
Good score (PASI ≥ 90)	45	7	13	25
Satisfactory score (PASI ≥ 75)	6	4	1	1
Negative effect of treatment				
Negative score (PASI ≤ 50)	30	16	13	1

^1^ The difference is significant compared with the etanercept group; ^2^ The difference is significant compared with the adalimumab group.

**Table 2:** Table of frequencies and minimum–maximum values of the studied cytokines in patients before and after treatment.

Cytokines	Percent (%) of Non-Zero Samples		Min–Max Values of Cytokines in Non-Zero Samples	
	Before	After	Before	After
IL1a	3.7	6.2	0.04–2.83	1.04–12.19
IL1b	14.8	49.4	0.01–0.57	0.02–0.87
IL4	49.4	17.3	3.71–217.46	0.7–72.28
IL6	14.8	27.2	0.21–231.97	0.02–42.94
IL10	29.6	24.7	1.36–320.33	0.09–93.88
IL11	6.2	7.4	0.3–2.16	0.05–7.58
IL12	44.4	69.1	0.34–10.0	0.07–14.62
IL17A	7.4	13.6	1.0–22.92	0.24–9.2
IL17F	48.1	21.0	9.0–245.55	5.23–59.03
IL20	28.4	54.3	0.55–65.05	0.09–94.22
IL21	17.3	7.4	9.0–366.11	3.72–39.27
IL22	33.3	9.9	0.3–175.54	0.25–41.23
IL23	16.0	1.2	0.71–173.34	2.24–2.24
IL25	49.4	25.9	0.04–9.0	0.05–7.23
IL31	88.9	70.4	7.78–867.5	2.9–241.71
IL33	43.2	13.6	25.41–419.5	0.65–300.63
TNFa	8.6	61.7	0.54–4.64	0.04–34.61
IFNg	2.5	8.6	6.91–49.58	2.19–83.85
ICAM1	100.0	100.0	149.15–878.4	2.63–668
sCD40L	100.0	97.5	14.58–3296.69	0.01–1763.96
VEGF	97.5	87.7	4.89–973.93	1.72–312.61

**Table 3:** Descriptive statistics of plasma cytokine levels in patients with moderate-to-severe psoriasis before and at week 16 of TNFα inhibitors therapy (only non-zero values are shown).

Cytokines	Before the Treatment with TNFα Inhibitors			After the Treatment with TNFα Inhibitors		
	Total Group (n = 81)	Positive Effect Group (n = 51)	No Effect Group (n = 30)	Total Group (n = 81)	Positive Effect Group (n = 51)	No Effect Group (n = 30)
IL1a	0.49 (0.27–1.66)	0.49 (0.27–1.66)	NA (NA-NA)	5.76 (4.38–6.08)	1.04 (1.04–1.04)	5.92 (5.415–7.61)
IL1b	0.22 (0.07–0.39)	0.19 (0.07–0.37)	0.27 (0.14–0.37)	0.2 (0.145–0.25)	0.22 (0.07–0.25)	0.19 (0.15–0.25)
IL4	34.40 (19.03–48.32)	34.4 (19.03–51.44)	35.34 (26.61–48.32)	13.47 (5.6–41.04)	26.41 (6.77–45.91)	7.86 (5.21–25.29)
IL6	26.83 (9.92–37.93)	30.58 (20.79–37.93)	14.54 (7.02–37.47)	4.14 (1.68–5.10)	2.09 (1.68–6.71)	4.37 (0.94–5.03)
IL10	15.24 (4.45–36.52)	11.63 (4.27–37.29)	20.48 (10.77–23.37)	15.89 (3.26–22.59)	4.845 (2.195–20.10)	19.955 (13.137–22.593)
IL11	1.48 (0.98–1.95)	1.72 (1.19–2.00)	0.98 (0.98–0.98)	2.88 (1.12–3.37)	3.32 (3.27–3.37)	1.59 (0.5–3.79)
IL12	2.01 (1.19–4.30)	1.93 (1.22–3.56)	2.98 (1.19–4.72)	2.12 (1.03–3.78)	2.06 (1.11–3.88)	2.18 (0.88–3.78)
IL17A	4.30 (1.47–8.45)	6.8 (4.30–14.86)	1.36 (1.18–5.18)	2.27 (0.57–3.57)	2.37 (2.32–4.11)	0.73 (0.43–3.09)
**IL17F**	**109.08 (55.21–177.06)**	67.66 (39.76–177.06)	111.03 (67.66–177.06)	**21.62 ** (12.09–27.09) *	21.48 (11.16–21.92)	25.51 (14.63–29.24)
**IL20**	16.17 (8.03–28.4)	**17.84 (10.46–30.23)**	**6.83 ** **(3.81–15.42) ^¶^**	7.92 (6.38–11.63)	8.16 (7.64–11.75)	7.64 (5.64–11.59)
IL21	52.22 (37.44–196.15)	52.26 (31.77–120.34)	52.22 (37.44–192.29)	19.39 (13.49–30.57)	22.91 (9.85–35.26)	19.39 (18.82–19.95)
IL22	7.64 (3.34–22.17)	5.5 (3.34–28.98)	8.32 (4.42–10.88)	2.52 (0.64–25.89)	1.29 (0.25–21.76)	3.75 (2.26–21.02)
IL23	72.49 (72.49–72.49)	72.49 (72.49–72.49)	72.49 (56.62–97.70)	2.24 (2.24–2.24)	2.24 (2.24–2.24)	NA (NA-NA)
IL25	2.78 (0.60–4.13)	2.21 (0.50–4.13)	4.13 (0.91–4.59)	0.38 (0.18–1.17)	0.475 (0.26–0.87)	0.38 (0.14–1.27)
**IL31**	**168.77 (70.401–310.04)**	**137.09 (56.39–293.38)**	**240.71 (137.09–327.88)**	**45.15** (24.51–81.16) *	**34.23 ** (23.35–64.89) *	**45.61 ** (25.93–81.18) *
IL33	108.57 (75.93–151.24)	101.96 (66.78–119.62)	140.86 (108.57–168.7525)	23.39 (9–86.15)	18.37 (12.76–33.79)	47.025 (8.3–139.86)
TNFa	1.04 (1.00–3.41)	2.21 (1.03–4.61)	1 (0.98–1.02)	4.61 (2.22–9.02)	4.07 (2.37–10.17)	4.96 (1.805–7.38)
IFNg	28.25 (17.58–38.91)	49.58 (49.58–49.58)	6.91 (6.91–6.91)	17.02 (6.50–33.69)	27.75 (13.81–49.82)	8.8 (5.495–18.85)
**ICAM1**	**261.27 (204.53–325.78)**	254.8 (203.01–328.95)	**268.57 (214.70–310.18)**	**318.34 ** (243.83–405.8) *	360.23 (256.97–456.49)	**311.47** (241.98–366.46) *
**sCD40L**	**444.14 (187.17–814.97)**	**431.05 (190.24–819.05)**	**466.01 (187.36–757.00)**	226.01 (57.1–475.39) *	**129.07 ** (49.19–465.74) *	306.44 (85.11–509.61) *
**VEGF**	**99.66 (49.48–205.25)**	**92.67 (53.02–213.32)**	**110.98 (28.37–198.12)**	**65.41 ** (26.57–131.6) *	**51.27 ** (12.66–101.02) *	**73.33 ** (30.24–139.13) *

* The difference is significant compared with the level before the treatment (highlighted in **bold**); ^¶^ The difference is significant compared with the positive effect group (highlighted in **bold**).

**Table 4:** Descriptive statistics of plasma cytokine levels in patients with moderate-to-severe psoriasis depending on the drug used (only non-zero values are shown).

Cytokines	Before			After		
	Etanercept (Enbrel)	Adalimumab (Humira)	Infliximab (Remicade)	Etanercept (Enbrel)	Adalimumab (Humira)	Infliximab (Remicade)
**IL1a**	NA (NA–NA)	0.49 (0.49–0.49)	1.435 (0.74–2.13)	4.38 (4.38–4.38)	3.56 (2.3–4.82)	8.975 (7.37–10.58)
**IL1b**	0.065 (0.0375–0.0925)	0.365 (0.25–0.4875)	0.16 (0.07–0.34)	0.18 (0.0825–0.215)	0.235 (0.145–0.25)	0.205 (0.15–0.3125)
**IL4**	34.4 (30.685–44.91)	26.97 (15.04–76.88)	34.4 (19.03–43.1975)	17.135 (7.5875–31.285)	33.825 (18.7675–48.8825)	13.1 (4.3075–35.575)
**IL6**	43.525 (21.8675–65.1825)	14.535 (7.0225–24.585)	30.58 (25.52–42.2625)	4.66 (3.635–5.685)	2.09 (1.68–3.615)	4.93 (0.94–5.12)
**IL10**	15.625 (10.77–22.6475)	11.63 (6.2–33.77)	18.85 (3.525–42.025)	11.34 (2.985–22.54)	4.2 (2.005–13.4075)	21.355 (14.67–23.3575)
**IL11**	NA (NA-NA)	0.98 (0.98–0.98)	1.715 (1.185–2.0025)	3.42 (3.42–3.42)	3.22 (3.22–3.22)	1.59 (0.5–3.7925)
**IL12**	1.25 (0.8–2.98)	3.65 (1.72–7.53)	1.965 (1.25–3.7575)	1.54 (0.87–2.98)	2.85 (1.65–3.85)	3.405 (1.9475–4.0875)
**IL17A**	9 (9–9)	1.18 (1.09–1.27)	6.8 (4.295–14.86)	5.85 (5.85–5.85)	2.27 (1.365–2.32)	0.78 (0.505–3.565)
**IL17F**	177.06 (67.66–177.06)	121.735 (53.4025–192.77)	67.66 (28.64–78.015)	21.34 (12.09–21.92)	18.125 (9.9425–21.845)	27.375 (22.3825–34.235)
**IL20**	6.83 (2.36–9.4)	16.17 (10.81–21.45)	18.62 (9.4–30.52)	7.92 (7.64–9.67)	7.64 (1.72–9.67)	10.7 (7.64–11.74)
**IL21**	37.44 (37.44–37.44)	67.07 (66.99–82.44)	124.41 (14.0525–267.065)	33.92 (18.82–36.595)	11.9 (11.9–11.9)	19.385 (18.8175–19.9525)
**IL22**	10.255 (5.9775–22.3175)	3.33 (2.22–4.42)	9.27 (3.34–29.16)	11.525 (6.4075–16.6425)	0.25 (0.25–0.25)	21.02 (3.005–39.025)
**IL23**	72.49 (72.49–72.49)	87.025 (43.8675–130.1825)	72.49 (72.49–72.49)	2.24 (2.24–2.24)	NA (NA-NA)	NA (NA-NA)
**IL25**	4.13 (2.84–4.5875)	0.855 (0.53–3.725)	2.66 (0.425–5.5025)	0.77 (0.38–0.97)	0.38 (0.22–0.57)	0.28 (0.135–1.67)
**IL31**	196.94 (132.55–270.46)	217.59 (120.5575–367.045)	120.03 (51.98–240.71)	44.215 (27.2–80.225)	45.15 (25.07–64.885)	44.42 (17.9625–84.5325)
**IL33**	108.57 (85.07–151.24)	101.96 (62.97–129.995)	108.57 (66.195–151.24)	59.93 (54.565–65.295)	12.76 (9.955–15.565)	23.39 (5.75–178.07)
**TNFa**	4.64 (4.64–4.64)	1.04 (1–1.625)	1.03 (0.785–2.82)	**9.2 (4.52–14.42)**	**2.17 (0.4675–2.9125) ^1^**	**2.82 (1.9–5.27) ^1^**
**IFNg**	6.91 (6.91–6.91)	NA (NA-NA)	49.58 (49.58–49.58)	17.02 (10.605–50.435)	23.64 (16.22–31.06)	15.545 (8.8675–22.2225)
**ICAM1**	245.16 (205.92–284.435)	251.65 (191.185–304.355)	299.69 (232.38–350.565)	318.68 (247.96–377.045)	318.34 (233.365–410.015)	312.22 (249.18–421.87)
**sCD40L**	317.66 (115.4–575.73)	479.94 (218.755–912.505)	450.75 (202.405–819.795)	187.64 (30.85–459.05)	288.16 (94.31–518.5)	228 (89.23–452.35)
**VEGF**	**75.875 (33.545–147.405)**	**84.4 (47.0775–219.2475)**	**152.31 (89.12–254.105) ^1^**	**31.445 (12.445–60.5575)**	**93.04 (32.385–134.605)**	**99.655 (48.4675–145.865) ^1^**

^1^ The difference is significant compared with the etanercept (Enbrel) group (highlighted in **bold**).

## Data Availability

The dataset analyzed during the current study is available from the corresponding author on reasonable request.
